# False Impressions

**Published:** 1889-02-09

**Authors:** Caroline Fothergill

**Affiliations:** Author of "An Enthusiast," "A Voice in the Wilderness," etc.


*(To be continued.)*

### FALSE IMPRESSIONS.

By Caroline Fothergill, Author of "An Enthusiast," "A Voice in the Wilderness," etc.

*(Continued from p. 288.)*

### Chapter XVIII.—The Last Straw.

Any doubts which Mrs. Lester might have had as to the legitimacy of using Edith as a means to draw her brother from his infatuation for Miss Stanford were dissipated by an incident which occurred immediately after the dinner party which had caused her such mental discomfort. It was not often she went out at all, still less often that she went out alone. On this occasion, however, she wished to take counsel with her particular friend Mrs. Hunter, and she wished her visit to remain unknown to her niece. Mrs. Lester was fond of indulging in actions which, although perfectly legitimate, it pleased her to keep shrouded in secresy. She got rid of Edith by sending her on an errand which would take her to quite a different part of the town from that in which she herself wished to go. Then she ordered the carriage and drove away. It was a fine afternoon, and contrary to custom she had the open carriage, the little Victoria which her brother often used.

Nothing happened during the drive to her friend's house. Her way lay through the most fashionable streets, and she saw people she knew whichever way she turned her head. Everyone was out, and this constant bowing and smiling quite restored her good humour.

She found Mrs. Hunter at home and alone, and as pleased to see her friend and gossip, Mrs. Lester, as that lady was to see her.

"And what have you come to tell me?" she asked, as they sat over their tea and cake.

"I came to ask how you thought Edith looked the other night," answered Mrs. Lester. "It was her first appearance at table, and I was particularly anxious she should appear to advantage; but I was really so occupied with other things I had no time to observe her. How did she look?"

Mrs. Hunter knew as well as Mrs. Lester herself that Edith's appearance and manner were not the chief causes of anxiety in that lady's mind, that this was only a little conversational skirmish preliminary to the real business in hand. But she knew equally well that in Mrs. Lester's position she would have gone to work in exactly the same way, so she entered at once into the spirit of the game.

"Edith looked charming," she pronounced, with the decision natural to her. "She was a little shy, perhaps, but that is a fault quite on the right side in a girl of her age, and if my eyes did not deceive me, her partner liked her all the better for it. Her dress was very pretty, and suited her exceedingly well; in fact, my dear, she is a wonderfully-pretty girl, and if she is going into society you will have to keep your eyes open, and be on the watch for matrimonial rocks ahead. That will be a totally new experience for you."

She laughed rather slyly as she spoke. Everyone knew what anxiety Mr. Murray caused his sister; and she had her own suspicion as to the reason for Mrs. Lester's call this day. She showed her wisdom, however, in letting her friend come to the point in her own fashion.

Mrs. Lester received this report on Edith with a look of pleasure.

"I am glad," she said. "After all, the girl is my niece, and I can't help being fond of her. I believe she is a good girl, and really grateful for all I have done for her."

"That is as it should be. I do not go out much, as you know, but I often hear of her being seen about with your brother. Is that so?"

"Yes, George is very kind to her; he has made her into quite a pet. I am only afraid she will get spoiled."

"That would be a great pity. Still, even more serious things than that might happen"—with a meaning smile.

"Oh, no," said Mrs. Lester easily. "I know what you mean, but you are quite mistaken. There is no danger in that direction. Edith knows her place too well, and George has no such thoughts."

"Ah, then you are at present free from anxiety on his account. I am glad of it, for his marriage would make a terrible change in your life, Frances."

Mrs. Lester was silent. She had admitted her fear to herself, but it was hard to have to confess it to someone else. Before she could make up her mind to speak, Mrs. Hunter went on—

"Of course, I should be the last to wish to suggest such a thing to you, and if I had been the only one to notice it I would not speak for the world. But since others saw it, too, I think it my duty, as your old friend, to put you on your guard."

Mrs. Lester still sat silent; chilled by the thought of what was coming, for she knew perfectly well what her friend was going to say. She had not been mistaken then—the spectre was indeed at her door. She rallied her courage, however, and said as indifferently as she could—

"You rouse my curiosity. I should be grateful if you would speak straight out."

"To be quite plain, then, my dear friend, a great many people were struck that evening with the attention your brother paid to Miss Stanford. His manner to her has raised a certain degree of speculation among the people who were at your house."

Mrs. Lester made no further effort at concealment. She scarcely waited for Mrs. Hunter to have finished speaking before she broke out—

"Then it was not my imagination! Other people did see it. That is what I came to ask."

Mrs. Hunter smiled at this admission, but generously forbare to point out the inconsistency in Mrs. Lester's speeches. She only said in a tone which held a note of pity—

"What made you commit such a mistake as to ask her to your house? It was not like you."

Mrs. Lester uttered that short laugh of scorn with which she was wont to greet all such ignorant and short-sighted statements.

"*I* ask her!" she repeated. "Do you suppose that I should ever have let such a woman cross my doorstep? I had very little choice in the matter. I was told to ask her, and in spite of all my protestations, which were as strong as I dared to make them, I had to yield in the end, as was only to be expected."

"Then Mr. Murray already knew her?" asked Mrs. Hunter, who was devoured with curiosity to hear all about this friendship which had created quite a sensation among Mrs. Lester's friends.

Mrs. Lester told all she knew, but as that was not nearly the whole of the history, her narrative left a good many gaps, which neither lady could satisfactorily fill up, as will be seen from the answer to the following question put by Mrs. Hunter.

"Then where did he first meet her?"

"At his museum. I always disliked the place. All these so-called charitable and philanthropic organisations are nothing but matrimonial hunting-grounds. I only wonder the men are so easily taken in."

Mrs. Hunter said nothing; she was thinking—

"Your brother is undoubtedly a great catch; but if all that I hear of Miss Stanford be true, she has no need to go 'slumming' to secure a husband. Such sweeping statements are a mistake." Aloud she said—

"And you had to call upon her?"

"George insisted upon it; and I found that she does not live alone, but has a kind of companion—a Mrs. Ledward, a widow, who is just as designing as herself, and as frivolous and light-minded as she can possibly be. Altogether——"

"Altogether it is rather a gloomy prospect," said Mrs. Hunter, as her friend paused.

"Well, yes," said Mrs. Lester; "I am afraid I must confess it is. At the same time I am not going to give up all hope. Everything is not lost yet, and of course I shall do everything in my power to prevent George from throwing himself away."

"Of course," echoed her friend. "Can I do anything to help you, dear friend?"

"No; I had better work it out alone. I am on the spot, and I know George best. I shall, no doubt, have a very anxious time until I see that things are going as I wish."

"I do not know Miss Stanford," said Mrs. Hunter. "I think I will call."

"If you do, I hope you may meet with a more friendly reception than I did," answered Mrs. Lester, with rather a grim laugh.

"How do you mean? Were you not well received? That seems strange."

"Oh—if my brother had been alone no doubt he would have had a very cordial greeting; but, you see, I was with him—an uninteresting old woman, a jealous sister. No doubt Miss Stanford had some way of making my brother understand that her coldness did not include him."

Mrs. Hunter only nodded, and Mrs. Lester, after sitting for a few minutes, as if wrapped in thought, rose and held out her hand.

"I must go," she said, "I always like to be at home first, and then George knows that when there is anything which he wishes to talk over with me, I am always there in my own sitting-room ready to listen to and sympathise with him."

Mrs. Hunter rose too. She grasped her friend's hand and kissed her cheek.

"Goodbye," she said. "You know that in whatever circumstances you may be placed you have my entire sympathy. Let me know how things go on. I shall think much of you, and shall feel very anxious."

She watched her friend drive away, exchanged a parting nod and smile with her, and then returned to her drawing room, her face wearing a very different expression from that which Mrs. Lester had seen.

"Poor Frances!" she murmured half aloud. "The end has come. Everyone sees it but yourself, and I suppose it is only natural that you should be blind. I prophesy that within six months your brother will be married, and married to Miss Stanford. Your place will know you no more, and if you are wise you will leave the town and settle quite at a distance."

Mrs. Lester felt better for her talk. On thinking over what had been said, she could not discover that Mrs. Hunter had uttered a single word calculated to dispel her fears. She had been kind and sympathetic, but she had expressed no conviction that Mrs. Lester was mistaken, and the lady's heart failed her. Nevertheless, she plucked up courage again, and made up her mind not to think of the desperate measure which had suggested itself to her until even plainer evidence than she had should be forthcoming. Mrs. Hunter's hint about Edith and Harry Fairfax returned to her, and she began to feel a little uncomfortable. Her good resolutions were destined all to be put to flight that moment.

She found, on looking at her watch, that it was later than she had thought, and she was afraid George would be home first, and that she would not be at hand to offer him consolation and sympathy in case he needed any. He never did, but she had begun with that rule and she intended to observe it as long as she lived in his house. She told the coachman to take a short cut to save time, and they turned out of the main street into a quieter narrow one. There were few people in it, and these were of a poorer class than in the street she had just quitted. Among the respectably dressed but undersized men and women walking along, two people of decidedly more than average height, and dressed as wealthy English gentlefolk are wont to dress were very conspicuous, and Mrs. Lester's eye leapt to them at once. She would have known her brother's figure and carriage in the densest crowd, and she identified without hesitation the erect queenly woman's figure at his side. Her heart seemed to contract, and for an instant her breath failed her. Then her pulses began to beat furiously again as she saw that they were coming towards her, that a meeting was inevitable. What should she do? Stop the carriage and speak to them? In the twinkling of an eye she had made up her mind that she would not do that, she would only bow and pass on. Nearer and nearer they came, and now she saw how absorbed they were in their conversation, whatever it might be about. Beatrice was speaking quickly, and apparently in some excitement. George was listening intently and eagerly, his eyes fixed upon her face, in evident unwillingness to lose one word of what she was saying. The subject of their conversation was quite free from mystery. A boy known to both of them had been guilty of gross abuse of his privilege to visit the museum, and his misconduct had been reported at a previous committee meeting. George was inclined to punish him severely, and make an example of him. Beatrice, thinking of the miserably unfavourable conditions under which the boy lived, was inclined to try persuasion in preference to force, and was taking advantage of an accidental encounter with George to plead the boy's cause eloquently.

This was the scene upon which Mrs. Lester came suddenly and unexpectedly; but she, knowing nothing of what they were speaking of, and by nature inclined to interpret intercourse between men and women in only one way, drew only one conclusion from their manner. Doubt was no longer possible. Her heart swelled, she felt her cheeks glow; and as the carriage met them, she drew herself erect, and gave them only the slightest and most formal recognition. Beatrice glanced up, and saw her, but George did not, and Mrs. Lester drove on with a feeling of death in her heart.

George noticed Beatrice's upward glance and slight inclination of the head, and asked impulsively—

"To whom were you bowing?"

"To your sister; we met her just now. Did you not see her?"

"No; how odd that she did not stop. Did she see us?"

"Oh, yes; she bowed."

"Perhaps she was in a hurry. About Ogden—you have convinced me that you are right, Miss Stanford. He shall be handed over to you, and you shall deal with him as you think fit."

"Thank you," she said, holding out her hand. "Good-bye."

So they parted, neither knowing what was to be the result of this meeting with Mrs. Lester.

